# Burnout syndrome among nephrologists - a burning issue – results of the countrywide survey by the Polish Society of Nephrology

**DOI:** 10.1186/s12882-020-01829-2

**Published:** 2020-05-12

**Authors:** Ewa Pawłowicz, Michał Nowicki

**Affiliations:** 1 Medical University of Lodz, Department of Nephrology, Hypertension and Kidney Transplantation, Pomorska Str. 251, 92-213 Lodz, Poland; 2 Polish Society of Nephrology , https://www.ptnefro.pl

**Keywords:** Burnout syndrome, Nephrologists, Nephrology

## Abstract

**Background:**

Burnout syndrome in physicians is associated with adverse patient safety events, poorer quality of care and reduced patients’ satisfaction. There has been scarce information on the risk factors of burnout affecting professionals working in the renal care settings. As yet the phenomenon has not been studied in the population of Polish nephrologists therefore a nationwide cross-sectional study was established by the Polish Society of Nephrology to assess the prevalence of the syndrome.

**Methods:**

The survey, that consisted of the abbreviated Maslach Burnout Inventory, questions about strategies for dealing with burnout symptoms and demographic data, was distributed during two main national meetings that gather nephrologists in Poland. 177 participants filled out the survey – 64% of participants were women, 88% were specialists and 12% - doctors in training.

**Results:**

52% of participants demonstrated a high level of depersonalization and almost half of the study group showed high level of emotional exhaustion. Reduced personal accomplishment was more pronounced in doctors working mostly in dialysis units compared to other nephrologists (*p* = 0.017). 37% of participants reported that they treat some patients as they were impersonal objects and 48% felt emotionally drained from their work. 59% of participants would like to take part in the remedy program.

**Conclusions:**

Burnout syndrome seems to be an important problem in the population of Polish nephrologists. Doctors working mostly in dialysis settings might be at increased risk of reduced personal accomplishment. The results of the survey may be useful to prepare burnout remedy program.

## Background

Burnout syndrome was first described by Herbert Freudenberger in 1974, an American psychologist who observed volunteers working in the free clinic for drug addicts in New York. Indeed, he ‘borrowed’ the term from the drug scene, which was colloquially used to address the final effects of a long-term drug abuse. Freudenberger described loss of motivation, emotional depletion and reduced commitment [1]. Throughout all his professional career Freudenberger focused mostly on the practical aspects of this phenomenon like preventing and combatting burnout rather than on scientific aspects. Simultaneously, since 1976, Christina Maslach from the University of Berkeley ran research on the same issue interviewing human service workers [2]. According to her studies burnout was defined as a three-dimensional syndrome consisting of interrelated emotional exhaustion, depersonalization and low personal accomplishment. Emotional exhaustion is defined as emotional depletion resulting from excessive job and/or personal demands and continuous stress [3], while depersonalization is understood as development of negative and cynical reactions to patients, as well as callous or even dehumanized perception, that can lead to view patients as somehow deserving of their troubles [4]. Reduced personal accomplishment is a tendency to evaluate oneself negatively, particularly with regard to one’s work with patients and dissatisfaction with their accomplishments on the job. Maslach and her team created a tool to measure burnout syndrome – a 22-item questionnaire – Maslach Burnout Inventory, which has become the most popular and most widely used survey in the psycho-sociological research related burnout syndrome [5].

It has been claimed in many studies that the work in health-care related fields is associated with increased risk of burnout syndrome. Shanafelt et al. interviewed more than 7000 participants of different professions and revealed that compared with other U.S. working adults, physicians were more likely to have the symptoms of burnout (38% vs 28%). Specialists at the front line of care i.e. family and emergency medicine doctors seemed to be at the greatest risk [6].

Investigating burnout syndrome among doctors is important not only due to its serious influence on the quality of life of a huge population of health-care providers, but mostly because of its meaning for patients’ safety and satisfaction. Tawfik et al., who performed a study among almost 6700 US physicians revealed that burnout and fatigue were independently associated with major medical errors [7]. Similarly, meta-analysis of 47 studies comprising more than 42,000 physicians from 19 countries all around the world showed that burnout was significantly associated with an increased risk of patient safety incidence, poorer quality of care due to decreased professionalism and reduced patient satisfaction [8], as well as decreased physicians’ productivity, assessed by number of sick leave days, work ability and intent to either continue practicing or change job [9]. These observations might be a reason why interest on burnout among researchers grows by the year. The number of publications of physician burnout indexed in PubMed database is growing rapidly – in 2018 almost 300 articles indexed with ‘burnout’ and ‘physician’ were published, while 60 articles from the period 1981–1989 are indexed in the database.

A reduction of burnout rates is necessary due to its influence on patients’ safety and satisfaction. However, asides from these reasons, a significant financial cost of burnout is raised. According to the recently published data by the group from the Stanford University, replacing a physician who resigns from his job because of burnout may cost at least $250,000. The awareness of this financial burden for the health-care system might be an additional motivation to invest money into preventing burnout [10].

Interestingly, more and more studies address the prevalence of burnout syndrome among residents and, noteworthy, medical students [11].

There are only few data available on burnout syndrome in the population of nephrologists. Recently, a short series of perspective articles discussing threats for nephrology as specialty, was published in Clinical Journal of American Society of Nephrology [12–15]. Burnout is raised as one of the most important, but still underestimated cause of declining interest and entry into training in nephrology. Attention is paid to complex actions aiming at recognizing, preventing and treating burnout among nephrologists.

Some data about burnout prevalence among US nephrologists is provided by the Medscape National Physician Burnout & Depression Report published annually [16, 17]. However, nephrologists comprised only 1% of the study sample in 2018 and 2019, and the groups numbered only around 150 physicians. The data published in 2018 and 2019 differed significantly in terms of percent of nephrologists reporting burnout – 40% vs. 32%, respectively.

There has been a paucity of data showing that some unique risk factors of burnout may occur in renal care professionals. Technologically advanced equipment, the intensive caring environment, long-term relationships being established between the caregiver and the patient as well as the chronic character of kidney diseases are presented as those, which may contribute to the development of burnout [18].

Nevertheless, we still do not know much about the prevalence and characteristics of burnout among nephrologists. Comparing to other specialties, e.g. oncology or emergency medicine, burnout in nephrology is a significantly underinvestigated phenomenon. Specifically, there are no studies that focused analysis on the differences in burnout among doctors working in dialysis and nephrologists who work in in-patient hospital settings. There were single reports that included only one of these groups [19]. We hypothesized that due to different characteristics of the professional care in these two renal settings the pattern of burnout may also differ.

Taking into account a lack of reliable data about burnout syndrome in the population of nephrologists and potential risk factors affecting this group, Polish Society of Nephrology (PTN) endorsed a nationwide cross-sectional study to investigate the prevalence and intensity of the phenomenon, what is crucial to design the prevention and remedy program. Besides that, a participation in such burnout-focused survey alone may encourage professionals to explore further the topic of burnout and to undertake some self-applied preventive measures.

## Methods

A 25-item paper-based questionnaires were distributed among participants of two largest national conferences that gather nephrologists – annual conference of the Polish Society of Nephrology in June 2018 and biannual Cracow Dialysis Days in September 2018. Every participant of the conference who was a practicing doctor was eligible for the study. Collection of the survey was announced by the chairpersons of the plenary sessions each day during these conferences. Surveys were placed on the chairs in the lecture halls and after the sessions participants were asked to get completed surveys to ballot boxes placed at all exits from lecture halls. Due to the method of data collection we could not prevent the same person filling out the questionnaire twice. However, we carefully checked the database to delete any duplicated demographic data. We have found only two pairs of records with overlapping demographic data and the duplicates were removed.

The study survey consisted of three parts. Part one was the abbreviated 9-item Maslach Burnout Inventory derived from the 22-item Maslach Burnout Inventory-Human Services Scale [20, 21]. The inventory comprised 3 statements referring to three dimensions of burnout. The respondents were asked to report the frequency of particular feeling associated with their job. Every statement was assessed on the time scale (never – 0 points, a few times a year – 1 point, once a month or less – 2 points, a few times a month – 3 points, once a week – 4 points, a few times a week – 5 points and everyday – 6 points). Adding the points of particular items allowed qualifying burnout in all three dimensions as low, moderate and high. The scoring for the particular dimensions was as follows: personal accomplishment: > 14 low, 13–14 moderate, < 13 high burnout; depersonalization: < 4 low, 4–6 moderate, > 6 high burnout and emotional exhaustion: < 7 low, 7–10 moderate, > 10 high burnout [21].

The second part included the questions about self-assessment of burnout, participation in the group or individual remedy programs as well as multiple-choice questions about self-applied strategies related to burnout prevention and relieving and self-assessed causes of burnout.

The third part of the survey comprised the questions on the demographic data and the work-related items. Questions about gender, age, time and level of professional experience, medical specialty, place of work, hours of work per week and the use of the last holiday leave were included. The participants were asked to mark all workplaces and indicate the main one. The second and the third parts of the study survey were developed by the authors. The study survey translated into English is provided as Supplementary File 1.

The study group comprised 177 physicians working in renal care settings regardless of their age and time of professional experience. According to the latest report of the Executive Committee of the Polish Society of Nephrology the Society counts 925 members. Our study group represents 19% of society’s members. The board-certified nephrologists, the specialists in internal medicine during nephrology training and the physicians in-training (residents) were involved in the study. The group characteristics are provided in Table 1.

**Table 1:** The characteristics of the study group of Polish nephrologists (*N* = 177)

Characteristic	Number of participants (%) or median (IQ range)
Gender	
men	64 (36.2%)
women	113 (63.8%)
Age	
< 30 years	15 (8.5%)
30–50 years	93 (52.5%)
51–62 years	59 (33.3%)
> 65 years	10 (5.7%)
Years of professional experience	20 (14–30)
Level of professional experience	
board-certified	156 (88.1%)
physician in-training	21 (11.9%)
Main place of work	
hospital (nephrology department)	100 (56.5%)
hospital (other department)	17 (9.6%)
nephrology out-patient clinic	3 (1.7%)
other out-patient clinic	1 (0.6%)
dialysis unit	56 (31.6%)
Number of workplaces	
1	50 (28.2%)
2	57 (32.2%)
3 and more	70 (39.6%)
Hours of work per week	
< 40 h	17 (9.6%)
41–50 h	50 (28.2%)
51–60 h	52 (29.4%)
61–75 h	40 (22.6%)
> 75 h	18 (10.2%)
Use of the holiday leave	
fully used	94 (53.1%)
partially used	77 (43.5%)
not used at all	6 (3.4%)

Statistical analysis was performed using Statistica version 13.1 PL software. Graphs were plotted with the use of Excel Office 365. The distribution of continuous variables was assessed with Shapiro-Wilk test. Mann-Whitney U test was used for comparisons between two independent groups. Nonparametric comparisons of more than two groups were performed with Kruskal-Wallis test. Chi^2^ test was used for comparisons of categorical data. Nonparametric correlations were assessed with Spearman’s method. Missing data in Maslach Burnout Inventory questions was 1.13% maximum. More missing data occurred in the demographic part of the survey, pairwise deletion was performed. To determine internal consistency reliability, Cronbach alpha was calculated for each dimension (3 items per dimension) of the Maslach Burnout Inventory. It amounted 0.82, 0.79 and 0.57 for emotional exhaustion, depersonalization and reduced personal accomplishment, respectively.

## Results

**Fig. 1:**
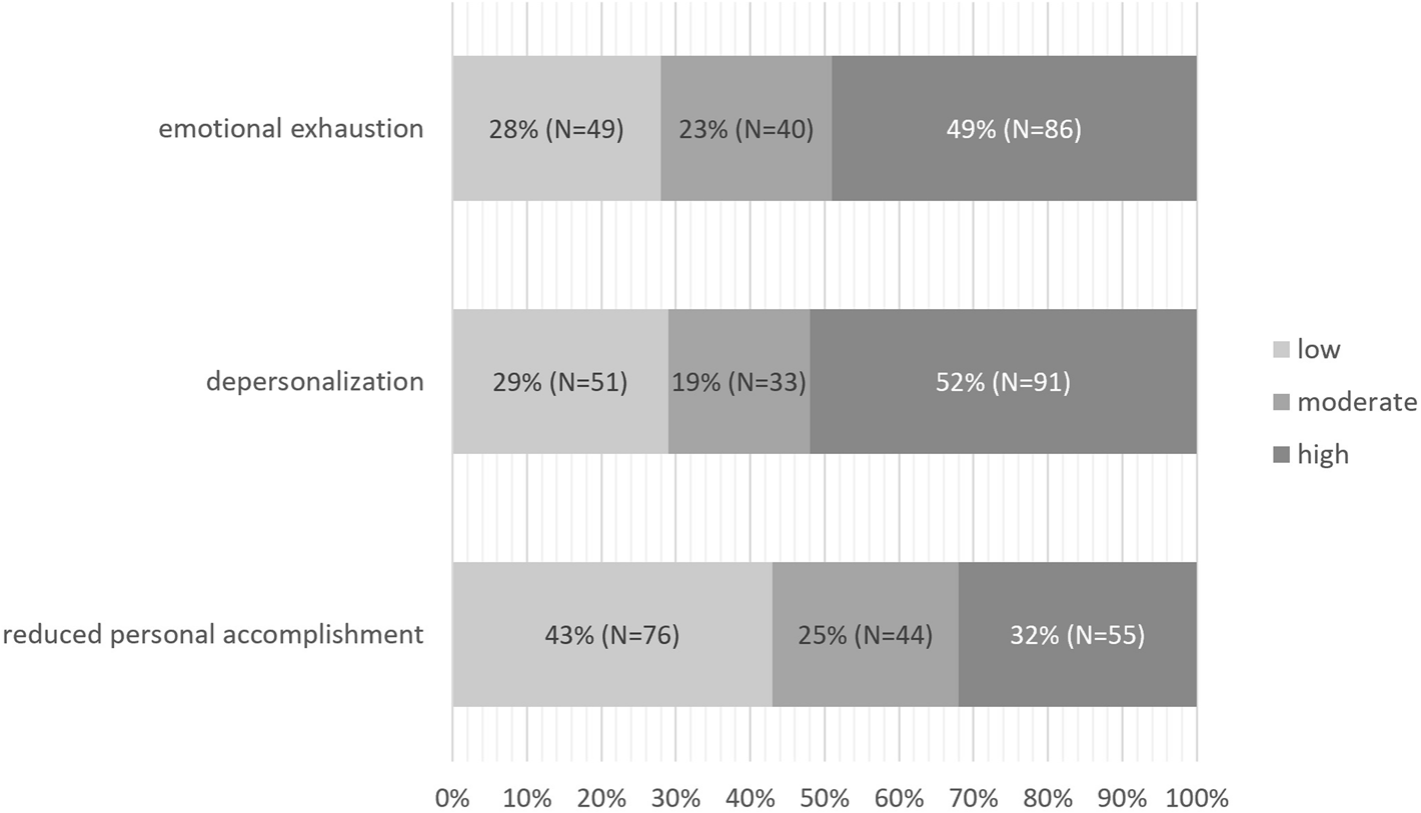
Percentage of Polish nephrologists experiencing low, moderate and high levels of burnout in particular dimensions

The results of the abbreviated Maslach Burnout Inventory are provided in Table 2. Median dimension score (IQ range) for reduced personal accomplishment, depersonalization and emotional exhaustion was 14 (12–16), 7 (3–10) and 10 (6–14), respectively. 48% of participants felt emotionally drained from their work at least once a week. 52% felt often fatigued already in the morning before another day on the job. Working with people all day was a real strain for 47% of studied physicians. 37% stated, that they treat some patients as impersonal objects at least once a week, and 36% has become more callous since they took their job. Only 14% stated that they do not care what happens to their patients at least once a week. At the same time, 93% reported that they deal very effectively with problems of their patients and 73.6% stated, that they are positively influencing their patients’ lives through their work. 64.1% felt exhilarated after working with their patients at least once a week. The intensity of burnout in particular dimension is showed in Fig. 1. 57, 71 and 72% of the study group suffered from at least moderate level of reduced personal accomplishment, depersonalization and emotional exhaustion, respectively. In 74 (42%) of the study participants at least moderate level of burnout was detected in all studied dimensions.

**Table 2:** Results of the abbreviated Maslach Burnout Inventory in the study group of Polish nephrologists; dimensions of burnout: RPA – reduced personal accomplishment, D – depersonalization, EE – emotional exhaustion

Item	Burnout dimension	N	Median score (IQ range)	N (%) of participants experiencing particular situation	
				Never	Every day
I deal very effectively with the problems of my patients.	RPA	177	5 (5–6)	2 (1.1%)	73 (41.2%)
I feel I treat some patients as if they were impersonal objects.	D	177	3 (1–5)	31 (17.5%)	8 (4.5%)
I feel emotionally drained from my work.	EE	175	3 (2–5)	14 (8.0%)	19 (10.9%)
I feel fatigued when I get up in the morning and have to face another day on the job.	EE	175	4 (2–5)	12 (6.9%)	23 (13.2%)
I’ve become more callous towards people since I took this job.	D	175	2 (1–5)	40 (22.9%)	12 (6.9%)
I feel I am positively influencing other people’s lives through my work.	RPA	175	5 (3–5)	2 (1.1%)	41 (23.4%)
Working with people all day is really a strain for me.	EE	176	3 (2–5)	21 (11.9%)	15 (8.5%)
I don’t really care what happens to some people I deal with at work.	D	175	1 (0–3)	72 (41.1%)	5 (2.9%)
I feel exhilarated after working closely with my patients.	RPA	175	4 (3–5)	5 (2.9%)	19 (10.9%)

Regarding sex differences, a high level of depersonalization was more common in men than in women (58% vs. 49%; *p* = 0.04; Chi^2^ test). No significant differences were found regarding reduced personal accomplishment and emotional exhaustion.

Interestingly, older physicians did not experience stronger burnout than younger doctors – median burnout intensity for all age groups was 7. The comparisons of age group in particular dimensions revealed that the emotional exhaustion was more pronounced in participants older than 30 years (*p* = 0.01; Chi^2^ test), no such finding was revealed in case of depersonalization and reduced personal accomplishment. The level of burnout experienced in particular dimension did not significantly depend on the years of professional experience.

High level of emotional exhaustion was more common among the board-certified specialists than among physicians in-training (52% vs. 29%; *p* = 0.01; Chi^2^ test), no differences in other dimensions were found.

Importantly, the comparison of burnout symptoms among doctors working mostly in dialysis centers and other participants revealed, that the former suffered more from the reduced personal accomplishment than other doctors, no such relation was found in case of other burnout dimensions (Table 3).

**Table 3:** Comparison of burnout intensity (median score (IQ range)) in three studied dimensions in Polish nephrologists working mostly in dialysis units and those working in other settings

Burnout dimension	Dialysis units *N* = 54	Other settings *N* = 117	*P* value^a^
Personal accomplishment	13 (11.5–15)	15 (12–16)	0.0175
Depersonalization	7 (4–10)	7 (2.5–10)	0.6757
Emotional exhaustion	11 (6.5–14)	10 (6–13)	0.6139

^a^ adjusted for sex, time of professional experience, hours of work/week and use of the holiday leave in the linear regression analysis

Hours of work and the use of the holiday leave did not influence burnout experienced by the participants.

Multiple linear regression analysis was performed with burnout dimension scores as dependent variables. Predictors taken into account were as follows: sex, time of professional experience, work place (dialysis/hospital), hours of work per week and the use of the last holiday leave. The only significant finding in this analysis was that reduced personal accomplishment was more pronounced in doctors working in dialysis centers. The logistic and linear regression models are provided as Supplementary File 2.

**Fig. 2:**
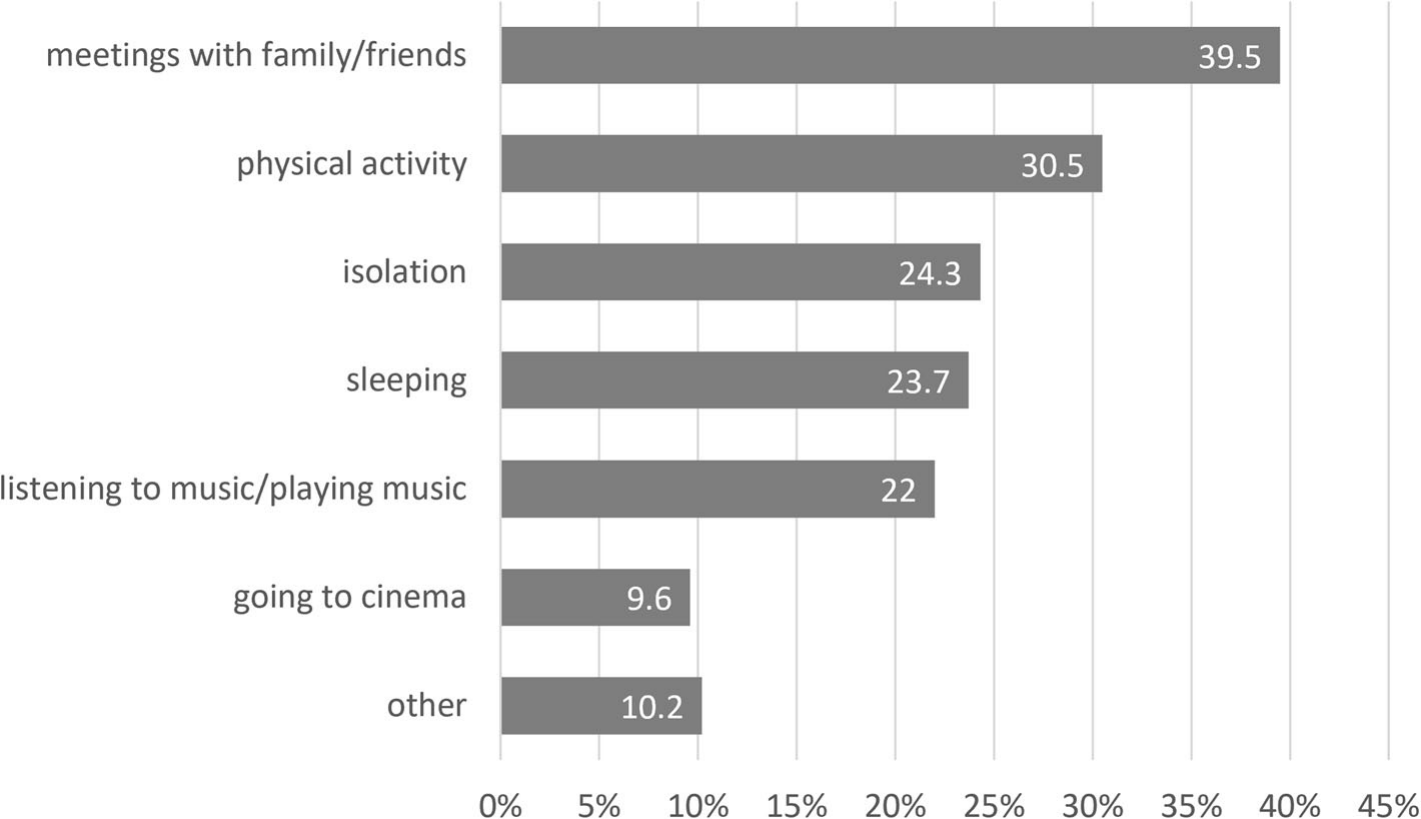
Self-applied strategies to deal with burnout symptoms in the studied group of Polish nephrologists

78 (44%) of participants answered ‘yes’ or ‘rather yes’ asked if they feel burnt out. Only 5 participants (3%) took part in burnout coping programs however 59% (*n* = 105) answered that they would like to participate in such program. 56% of them preferred group rather than individual coping programs. 28% of the participants who would like to take part in such program thought that the program should be obligatory for the physicians with the diagnosed or suspected burnout syndrome and it should be offered and covered by the employer. 70% stated that such the program should be voluntary and provided by the employer. 79% of the study participants stated that they take some steps towards relieving of burnout symptoms. These self-applied strategies of coping with burnout are provided in Fig. 2.

**Fig. 3:**
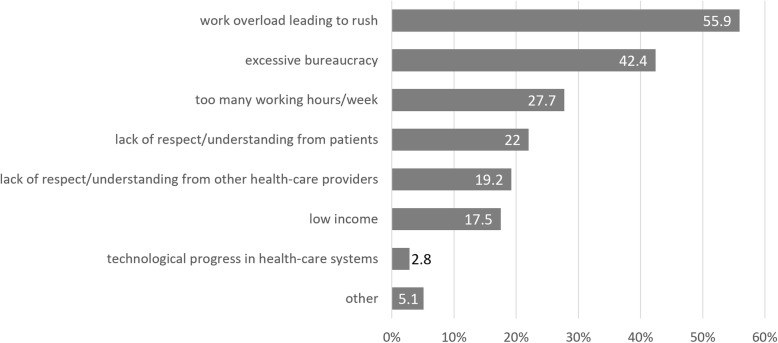
Percent of Polish nephrologists indicating particular work-related factor which may contribute to development of burnout symptoms

The participants were asked also about work-related factors which may contribute to development of burnout symptoms. Most of them stated that work overload leading to rush was the most aggravating factor (Fig. 3). Among ‘others’ participants enumerated i.e. a lack of workforce and lack of sufficient amount of equipment.

## Discussion

Physicians’ burnout was described as a crisis in health care in the Physician Burnout Report 2018 [22]. The authors indicated that in the U.S. population the prevalence of burnout has reached a critical level with nearly half of all physicians experiencing burnout in some form.

According to our report burnout syndrome seems to be a serious problem also in the population of Polish nephrologists, mostly in terms of emotional exhaustion and depersonalization, which high levels were observed in the majority of the study group. The doctors working mainly in dialysis units struggled more with the problem of reduced personal accomplishment than their colleagues, who worked mostly in the nephrology in-patients hospital settings. It may be related to the poor prognosis of patients on chronic hemodialysis [23], a significant comorbidity in this population [24] and a low adherence to medical recommendations especially in terms of diet and fluid restriction [25]. The work in dialysis units is considered as the most aggravating part of the career in nephrology. Lane and Brown performed a qualitative analysis of the perceptions of nephrology among trainees facing a career choice. Young doctors raised the complexity of medical problems, poor prognosis and compliance of patients suffering from end-stage renal disease, especially those on dialysis. ‘Hopelessness’ was a main theme identified regarding the work in the dialysis units [26]. It might be hypothesized that the similar perception may also occur in some already practicing nephrologists. Sharif et al. provided the evidence that the global nephrology workforce failed to expand in face of the growing healthcare needs associated with the rising burden of chronic kidney disease worldwide [27]. Any factor that could lead to even lower rate of young physicians choosing career in nephrology or practicing doctors leaving from work should be taken into careful consideration. One of such factors might be burnout symptoms.

Practicing nephrology is associated with some specific and unique circumstances that may increase the risk of burnout syndrome. One of the most important might be the complexity of renal patients. The recently published data showed that the nephrologists deal with the most complex patients, comparing to other 12 studied specialties [28]. However, as we take into consideration the scientific publications about burnout prevalence in the particular medical domain, this specialty is rarely addressed, giving room mostly to oncology, emergency medicine and intensive care.

Interestingly, there is significantly more information on burnout syndrome among nurses than doctors working in the renal care settings [29–35].

To the best of our knowledge, there has been only a single original study considering burnout syndrome among nephrologists working in dialysis units. Argentero et al. on behalf of the Working Group on Burnout and Dialysis examined nephrologists’ and nephrology nurses’ burnout together with patients’ satisfaction rates. They showed that high levels of burnout in renal physicians and nurses were associated with poor patients’ satisfaction in dialysis setting [19]. The study, performed in 10 dialysis centers in northern Italy, comprised only 68 physicians compering to 334 interviewed nurses, and, according to the authors, physicians were less emotionally involved in patients’ problems than nurses.

As mentioned above, burnout syndrome has been observed even among medical students [36] and residents [37], so the prevalence of burnout cannot be simply associated with the job seniority. Our data suggest, that the age and the level of professional experience (board-certified specialists vs. in-training doctors) are associated with higher emotional exhaustion, but this relation was not observed in case of personal accomplishment and depersonalization.

A coping with burnout symptoms has become a very important challenge nowadays. As pointed out in our study, there is a huge need for burnout prevention and remedy programs among Polish nephrologists. It might be an encouraging finding, since it may indicate that the physicians are actively looking for possible opportunities of participation in such programs. Currently, there are more and more remedy and prevention programs in Poland provided mostly by local chambers of physicians and dentists. Within the Local Chamber of Physicians and Dentists in Warsaw a Special Team on Burnout was established in 2017. The team is responsible for providing a professional and free of charge help for doctors suffering from burnout [38]. Besides that, the Balint groups become more and more popular in Poland. Balint group method is focused mostly on discussing a patient-doctor relationship and providing the peer-support. There is the evidence that the participation in Balint group may relieve burnout symptoms [39]. It should be emphasized, that there is a need to increase awareness about burnout and its significance for a daily practice among doctors, what could contribute to more common participation in the provided coping programs.

In our study the work overload leading to rush was the most important factor that contributed to burnout development. The issue of work overload and organizational insufficiency of Polish health-care system is beyond the scope of this article. The second most reported cause among Polish nephrologists was the excessive bureaucracy in the daily practice. Too many bureaucratic tasks was revealed as the most important burnout factor in the Medscape Global Physicians’ Burnout and Lifestyle Comparisons project [40]. In the report data collected from 20,000 physicians from six countries - USA, UK, Germany, Spain, Portugal and France were taken into account. In 5 of 6 analyzed countries excessive bureaucracy was reported most often as burnout increasing factor, it was raised by 56% of US and Portuguese doctors, 52, 49 and 47% of German, French and UK physicians, respectively. Only Spanish respondents indicated low income as the most important burnout factor.

A collection of the survey during the nephrology national conferences might be considered as a limitation of our study since that may lead to the selection bias leading to limited generalizability. It might be assumed that the conferences participants are more successful in their career and thus less depressive and burned out. On the other hand, the polish nephrologists is a relatively small population and many of the practicing nephrologists, also practicing in smaller health-care centers participate in educational meetings. Also, the most aggravating factors related to everyday practice might be more likely reported as measured in the same environment (hospital, dialysis unit) than during the conference. The significantly larger group of board-certified specialists than physicians in-training in the studied population may impede comparisons of these two groups. Besides that, social desirability bias should be taken into account, especially regarding items questioning whether physician treats patients as impersonal objects or is callous towards people.

Among the significant strengths of our work is a large sample size of 177 physicians, to the best of our knowledge the most numerous group of renal care settings doctors interviewed on burnout as yet. It may be compared to Medscape Reports, where around 150 nephrologists were surveyed in U.S. nationwide study. Another important advantage of our research is the separation of two subgroups from the study population i.e. the doctors working mostly in dialysis units and those who work mainly in other settings.

## Conclusions

Our study is the first nationwide research on burnout among polish nephrologists, that gives an insight into the prevalence and intensity of burnout syndrome in this group of physicians. Besides, we provided the evidence that the pattern of burnout symptoms’ differs between doctors working in dialysis settings and physicians working mostly in hospitals. The qualitative research in this field could contribute to deeper understanding of this phenomenon. Our findings might be helpful in creating remedies and prevention programs, that will focus on the most affected aspects of doctors’ practice.

## Supplementary information

**Additional file 1.** The study survey – the full English language version.

**Additional file 2.** Additional analysis results – logistic and linear regression models.

## Data Availability

The dataset analysed during the current study is available from the corresponding author on request.

## Abbreviations

RPA: Reduced personal accomplishment
D: Depersonalization
EE: Emotional exhaustion
