# Supplementary material for: Burnout syndrome among nephrologists - a burning issue – results of the countrywide survey by the Polish Society of Nephrology
Source: BMC Nephrol. 2020 May 12;21:177. doi: 10.1186/s12882-020-01829-2 (PMC7218642; doi:10.1186/s12882-020-01829-2)
Supplement: Supplementary file 1 — Additional file 1. The study survey – the full English language version. [file 12882_2020_1829_MOESM1_ESM.docx]

Dear Participant,

On behalf of the Executive Committee of the Polish Society of Nephrology we would like to ask you to take part in the survey aiming at measuring and describing the phenomenon of burnout in the population of nephrologists and other specialties' doctors as well as doctors-in-training working in nephrology and dialysis settings in Poland.

It was already confirmed in many studies that burnout may significantly affect physicians' practice and clinical performance. Nephrologists may be at the particular risk of burnout due to chronic character of kidney diseases and dialysis-related issues.

There are some data about prevalence of burnout in the population of US nephrologists, but little is known about burnout syndrome among polish renal physicians.

Proper measuring and assessing burnout syndrome is a key to success in creating adequate remedy programs.

The survey is completely anonymous, and analyzed data will be used scientifically and for creating the burnout remedy program responding for actual needs.

By completing the survey, participants are stating consent to participate in the study.

Thank you in advance for taking part in the project.

Best regards,

Prof. Michał Nowicki MD, PhD

*President of the Polish Society of Nephrology*

Ewa Pawłowicz, MD

*Young Nephrologists’ Club of the Polish Society of Nephrology*

**Burnout syndrome assessment (abbreviated Maslach Burnout Inventory)**

|  | Every day | A few times a week | Once a week | A few times a month | Once a month or less | A few times a year or less | Never |
| --- | --- | --- | --- | --- | --- | --- | --- |
| I deal very effectively with the problems of patients. |  |  |  |  |  |  |  |
| I feel I treat some patients as if they were impersonal objects. |  |  |  |  |  |  |  |
| I feel emotionally drained from my work. |  |  |  |  |  |  |  |
| I feel fatigued when I get up in the morning and have to face another day on my job. |  |  |  |  |  |  |  |
| I've become more callous toward people since I took my job. |  |  |  |  |  |  |  |
| I feel I am positively influencing other people's lifes through my work. |  |  |  |  |  |  |  |
| Working with people all day is really a strain for me. |  |  |  |  |  |  |  |
| I don't really care what happens to some patients. |  |  |  |  |  |  |  |
| I feel exhilarated after working closely with my patients. |  |  |  |  |  |  |  |

**Dealing with burnout syndrome**

1. Do you feel burned out?

yes

rather yes

rather no

no

2. Do you participate now or have you ever participated in the burnout remedy or/and prevention program?

yes no

3. If no, would you like to participate in burnout remedy or/and prevention program?

yes no

4. If so, such program should be:

group meetings

individual meetings

5. If so, such program should be:

obligatory for those who were diagnosed with burnout syndrome, founded by the employer

voluntary, but founded by the employer

voluntary, financed by the own resources of the doctor

6. Do you apply any strategies to combat burnout on your own?

no yes

7. If so, what do you do?

physical activity

meetings with family/friends

listening to/playing music

sleeping

isolating myself from others

going to cinema

other, please specify.............................................

8. What contributes most to your burnout? [indicate all fitting options, but avoid to tick all provided options]?

too many bureaucratic tasks

spending too much time at work

lack of respect from administrators/employers/staff/colleagues

lack of respect from patients

work overload leading to rush

increasing computerization of practice

insufficient compensation

other, please specify..............................................................................................................

**Work-related and demographic data**

1. Gender:

female male

2. Age:

<30 years old

30-50 years old

51-65 years old

> 65 years old

3. Years of professional experience:

…………………… years

4. I am:

nephrologist (board-certified specialist)

internal medicine specialist in-training in nephrology

in-training doctor/resident (in internal medicine)

in-training doctor/resident (in nephrology)

other, please specify…………………………………………………………....

6. I work in: [mark only one place that is your main workplace e.g. you spend most hours]

- hospital (nephrology ward)
- hospital (other ward)
- out-patient clinic (nephrology)
- out-patient clinic (other)
- dialysis unit
- emergency department

5. I work in: [mark every setting that fits]

hospital (nephrology ward)

hospital (other ward)

out-patient clinic (nephrology)

out-patient clinic (other)

dialysis unit

emergency department

6. How many hours per week do you spend at work?

not more than 40 hours

41-50

51-60

61-75

more than 75 godzin

7. Did you take your holiday leave last year?

yes, I used all days

yes, partially

not at all
