# Supplementary material for: Burnout syndrome among nephrologists - a burning issue – results of the countrywide survey by the Polish Society of Nephrology
Source: BMC Nephrol. 2020 May 12;21:177. doi: 10.1186/s12882-020-01829-2 (PMC7218642; doi:10.1186/s12882-020-01829-2)
Supplement: Supplementary file 2 — Additional file 2. Additional analysis results – logistic and linear regression models. [file 12882_2020_1829_MOESM2_ESM.docx]

**Supplementary file 2.**

**Additional analysis results – logistic and linear regression models.**

We performed linear regression analysis with burnout scores in each dimension as dependent variables. Predictors taken into account were as follows: sex, time of professional experience, work place (dialysis/other settings), hours of work per week and the use of the last holiday leave.

Besides, we performed logistic regression analysis with at least moderate intensity in all studied dimensions as dependent variable. The model turned out not to be significant.

**Logistic regression model**


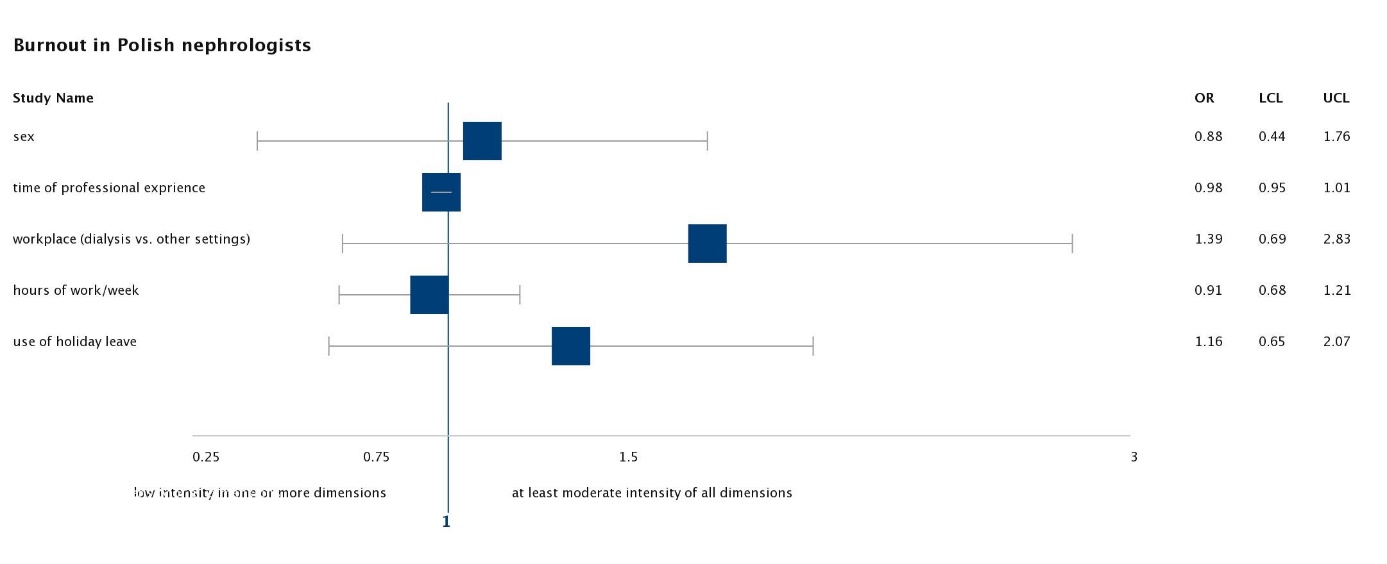


| Chi-Square=3.2065 | df=5 | p-value=0.6682 |
| --- | --- | --- |

| **Variable** | **Coefficient** | **Standard Error** | p**-value** | **Odds Ratio** | **95% Confidence Interval** |  |
| --- | --- | --- | --- | --- | --- | --- |
| Sex | -0.1254 | 0.3510 | 0.7209 | 0.8822 | (0.4434,1.7552) |  |
| time of professional experience | -0.0183 | 0.0146 | 0.2088 | 0.9818 | (0.9542,1.0103) |  |
| workplace (dialysis vs. other settings) | 0.3317 | 0.3606 | 0.3577 | 1.3933 | (0.6872,2.8251) |  |
| hours of work/week | -0.0956 | 0.1471 | 0.5160 | 0.9089 | (0.6812,1.2126) |  |
| use of the holiday leave | 0.1460 | 0.2978 | 0.6239 | 1.1572 | (0.6456,2.0743) |  |
| Constant | 0.2072 | 0.8022 | 0.7962 |  |  |  |

**Linear regression model for Reduced personal accomplishment**

**reduced personal accomplishment**=13.4283−0.2171⋅sex+0.0252⋅time of professional experience−1.1857⋅workplace (dialysis vs. hospital)+0.132⋅hours of work/week−0.0462⋅use of the holiday leave

**overall p value 0.1733**

| **Predictor** | **Coefficient** | **Estimate** | **Standard Error** | p**-value** |
| --- | --- | --- | --- | --- |
|  |  |  |  |  |
| Constant | β0 | 13.4283 | 1.0906 | 0 |
|  |  |  |  |  |
| sex | Β_1_ | -0.2171 | 0.4758 | 0.6489 |
|  |  |  |  |  |
| time of professional experience | β2 | 0.0252 | 0.0196 | 0.2 |
|  |  |  |  |  |
| workplace (dialysis vs. other settings) | β3 | -1.1857 | 0.4936 | **0.0175** |
|  |  |  |  |  |
| hours of work/week | β4 | 0.132 | 0.1988 | 0.5076 |
|  |  |  |  |  |
| use of the holiday leave | β5 | -0.0462 | 0.4056 | 0.9095 |
|  |  |  |  |  |

**Linear regression model for Depersonalization**

**depersonalization**=4.8582+0.2064⋅sex−0.0154⋅time of professional experience+0.3481⋅workplace (dialysis vs. hospital)+0.1901⋅hours of work/week+0.8746⋅use of the holiday leave

**overall p value 0.7221**

| **Predictor** | **Coefficient** | **Estimate** | **Standard Error** | p**-value** |
| --- | --- | --- | --- | --- |
|  |  |  |  |  |
| Constant | β0 | 4.8582 | 1.8353 | 0.009 |
|  |  |  |  |  |
| sex | β1 | 0.2064 | 0.8007 | 0.797 |
|  |  |  |  |  |
| time of professional experience | β2 | -0.0154 | 0.033 | 0.6407 |
|  |  |  |  |  |
| workplace (dialysis vs. other settings) | β3 | 0.3481 | 0.8305 | 0.6757 |
|  |  |  |  |  |
| hours of work/week | β4 | 0.1901 | 0.3345 | 0.5706 |
|  |  |  |  |  |
| use of the holiday leave | β5 | 0.8746 | 0.6825 | 0.202 |
|  |  |  |  |  |

**Linear regression model for Emotional exhaustion**

**emotional exhaustion**=8.2921−0.5509⋅sex+0.012⋅time of professional experience+0.4179⋅workplace (dialysis vs. hospital)+0.1468⋅hours of work/week+0.9991⋅use of the holiday leave

**overall p value 0.634**

| **Predictor** | **Coefficient** | **Estimate** | **Standard Error** | **-value** |
| --- | --- | --- | --- | --- |
|  |  |  |  |  |
| Constant | β0 | 8.2921 | 1.8267 | 0 |
|  |  |  |  |  |
| Sex | β1 | -0.5509 | 0.797 | 0.4905 |
|  |  |  |  |  |
| time of professional experience | β2 | 0.012 | 0.0328 | 0.7152 |
|  |  |  |  |  |
| workplace (dialysis vs. other settings) | β3 | 0.4179 | 0.8266 | 0.6139 |
|  |  |  |  |  |
| hours of work/week | β4 | 0.1468 | 0.3329 | 0.6598 |
|  |  |  |  |  |
| use of the holiday leave | β5 | 0.9991 | 0.6793 | 0.1434 |
|  |  |  |  |  |
